# Higher-Order Thermo-Elastic Analysis of FG-CNTRC Cylindrical Vessels Surrounded by a Pasternak Foundation

**DOI:** 10.3390/nano9010079

**Published:** 2019-01-08

**Authors:** Masoud Mohammadi, Mohammad Arefi, Rossana Dimitri, Francesco Tornabene

**Affiliations:** 1 Department of Solid Mechanics, University of Kashan, Kashan 87317-51167, Iran; m.mohammadi.academic@gmail.com; 2 Department of Innovation Engineering, University of Salento, 73100 Lecce, Italy; rossana.dimitri@unisalento.it

**Keywords:** carbon nanotubes, composite cylindrical pressure vessel, functionally graded materials, third-order shear deformation theory

## Abstract

This study analyses the two-dimensional thermo-elastic response of functionally graded carbon nanotube-reinforced composite (FG-CNTRC) cylindrical pressure vessels, by applying the third-order shear deformation theory (TSDT). The effective properties of FG-CNTRC cylindrical pressure vessels are computed for different patterns of reinforcement, according to the rule of mixture. The governing equations of the problem are derived from the principle of virtual works and are solved as a classical eigenproblem under the assumption of clamped supported boundary conditions. A large parametric investigation aims at showing the influence of some meaningful parameters on the thermo-elastic response, such as the type of pattern, the volume fraction of CNTs, and the Pasternak coefficients related to the elastic foundation.

## 1. Introduction

Pressure vessels are one of the most important and expensive components for mechanical and structural engineering applications. Due to their importance, some international companies have published useful guidelines for design purposes, e.g., the ASME Standards [1]. At the present stage, however, the available standards do not deliver any suggestion for the design of reinforced pressure vessels. To this end, the structural behavior of reinforced pressure vessels has gained increasing attention among the scientific community, as detailed in what follows.

A preliminary work was proposed by El Mir et al. [2] to analyze cylindrical sandwich structures made of a weak orthotropic core under a certain patch loading. The authors presented a novel numerical formulation based on a high-order shear deformation theory (HSDT) to predict the structural response in static conditions.

Arefi and Rahimi [3] and Arefi et al. [4,5,6] studied the thermo-elastic behavior of non-homogeneous functionally graded (FG) cylinders with clamped supports under a double mechanical and thermal loading condition, while applying the first-order shear deformation theory (FSDT). Menasria et al. [7] employed an innovative displacement field with unknown integral terms to analyze the thermal buckling behavior of a FG sandwich plate under a uniform thermal loading. They derived the governing equations of the problem, based on a variational principle. A further work by Dong et al. [8] studied the local buckling behavior of composite plates resting on a Wrinkler foundation under a uniform in-plane shear loading, while focusing on the effect of the ply angle and stiffness foundation on the critical buckling coefficients.

Ahmad Bidi et al. [9] studied a reinforced curved steel structure with nanopolyurea, under a low velocity impact loading. A double experimental and numerical study was performed by the authors to explain the effect of the in-plain curvature and the nano-particle effect on the structural response. In a recent work by Rahmani et al. [10], the Hamilton’s principle and the Navier solution were applied to examine different non-local HSDTs for FG nanobeams, including the size-dependent effects. Golmakani et al. [11], in addition, studied the non-linear thermo-elastic bending behavior of functionally graded carbon nanotube-reinforced composite (FG-CNTRC) plates resting on an elastic foundation. Nasihatgozar et al. [12] used a HSDT to study the free vibration response of thick-layer doubly curved sandwich panels resting on simply supports. This investigation delivered an optimum range for the core to face sheet stiffness ratio by considering the effective stress components, for the first time.

Arefi et al. [13] investigated the thermo-piezo-magneto-elastic behavior of a FG piezomagnetic cylinder under thermal, magnetic and mechanical loading, which was, in turn, FG in the radial direction. Mohammadimehr et al. [14] considered a viscoelastic piezoelectric polymeric nano-composite plate reinforced by FG single-walled carbon nanotubes (SWCNT) based on a modified strain gradient theory. The authors, in their work, studied the effect of the thickness-to-width ratio, as well as of the magnetic field, the applied voltage, the static loading factor, the viscoelastic parameters of the foundation, and the surface density constant, on the dynamic stability region. In addition, Hao et al. [15] focused on the thermo-mechanical stresses within the graded interphase between the fiber and the matrix in fiber-reinforced composites. Shen et al. [16] presented a non-linear bending analysis for nanocomposite cylindrical panels subjected to a transverse uniform or sinusoidal load resting on an elastic foundation in a thermal environment. They studied the effect of the CNT volume fraction distribution, the foundation stiffness, the temperature rise and the in-plane boundary conditions on the mechanical response of the structure. Moreover, Arefi and Rahimi [17] applied the FSDT to study the electro-elastic response of a FG piezoelectric cylinder under internal pressure.

Alibeigloo [18] investigated the free vibration behavior of FG-CNTRC cylindrical panels embedded in piezoelectric layers with simply supported boundary conditions. A coupled thermo-mechanical problem was also treated by Shen and Xiang [19], who considered the nanocomposite cylindrical shells reinforced by SWCNTs in thermal environments and studied the sensitivity of the response in terms of natural frequencies as well as of non-linear-to-linear frequency ratios for varying temperatures and CNT volume fractions. In line with the previous work, Arefi et al. [6] presented a two-dimensional thermo-elastic analysis of a FG thick-walled cylinder under thermal and mechanical loading resting on a Pasternak foundation. They applied the FSDT to describe the displacement field and found that an increased non-homogeneous index yields to a decrease of the radial and axial displacement. A finite element method (FEM)-based numerical analysis was applied by Chavan and Lal [20] to analyze the static behavior of SWCNT-FG plates. A large parametric investigation was aimed at investigating the effect of the width-to-thickness ratio, the stress distribution profile along the thickness, the boundary conditions, as well as the volume fractions on the overall structural behavior.

A further coupled thermo-piezoelectric problem was proposed by Khoshgoftar et al. [21], for a thick-walled cylinder with FG material, subjected to an inner and outer pressure under a constant temperature gradient. An interesting post-buckling coupled problem can be found in Kiani [22], who studied the thermo-mechanical behavior of CNTRC plates under a uniform increase of the thermal loading, and determined the effect of the reinforcement pattern on the critical temperature and the maximum post-buckling deflection for different distributions. Zhang et al. [23] investigated the free vibration response and flexural strength of CNTRC cylindrical panels with four different reinforcement distributions. Moreover, Rahimi et al. [24] focused on the electro-elastic behavior of a FG piezoelectric cylinder adopted as a physical sensor, and subjected to an internal pressure. A further work by Asadi et al. [25] aimed at investigating the aero-thermo-elastic behavior of supersonic FG-CNTRC flat panels in a thermal environment. To this end, a dynamic model was developed by the authors, according to the FSDT, whereas the presence of an aerodynamic pressure was found to play a key role not only for the onset of aero-thermal buckling instability, but also for the mode shapes of the composite structure.

In recent years, increased attention has been devoted by the scientific community to a comparative evaluation of FSDTs and HSDTs in a combined form with the novel generalized differential quadrature (GDQ) method for the study of the statics and dynamics of composite plates and shells of arbitrary shapes reinforced by agglomerated nanoparticles (see [26,27,28,29,30,31,32,33,34,35] among others). Despite the available works in the literature on the reinforced cylindrical pressure vessels, there is a general lack of analytical formulations, based on the third-order shear deformation theory (TSDT), to study FG-CNTRC cylindrical pressure vessels, in thermal conditions. In this context, the main task of the present work regards the novel application of the TSDT to study the thermo-mechanical response of a two-dimensional FG-CNTRC cylindrical pressure vessel resting on a Pasternak foundation. This represents an extended version of the FSDT-based formulation proposed in Arefi et al. [6] for the same problem, whose governing equations were tackled as a classical eigenproblem, for a clamped structure at the two extremities. In this work, a parametric analysis aims at investigating the sensitivity of the thermo-mechanical response of the composite structure to some important parameters such as the reinforcement pattern, the volume fraction of CNTs, or the Pasternak parameters related to the foundation. This represents a useful aspect to account for practical industry applications and optimization design. The paper is organized as follows. First, we present the problem formulation in Section 2, whose solution procedure is briefly reviewed in Section 3. Next, the parametric investigation and the main results are presented and discussed in Section 4. Finally, conclusions are drawn in Section 5.

## 2. Problem Formulation

### 2.1. Geometry and Material Properties

**Figure 1:**
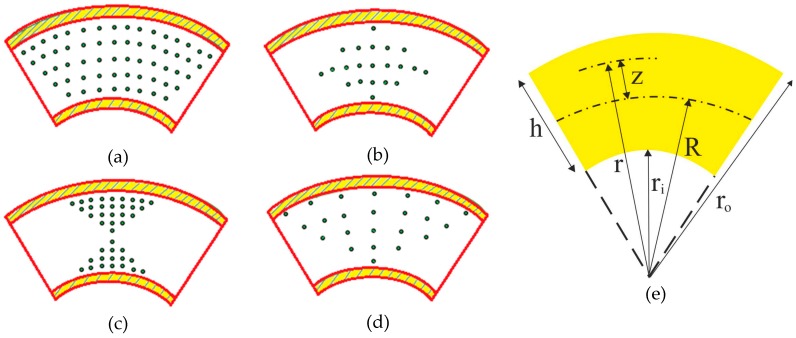
Geometry of the carbon nanotube-reinforced composite (CNTRC) cylindrical shell, with different CNTs distribution patterns: (**a**) UD, (**b**) FG-O, (**c**) FG-X, (**d**) FG-V, (**e**) Geometry.

Let us consider a FG cylindrical shell with geometrical parameters defined in Figure 1. It is possible to assume different CNT distributions along the thickness, whose geometries are depicted in Figure 1, with the analytical expressions reported in Table 1 as a function of the effective volume fraction $V_{CNT}^{*}$, in which R, r and h indicate the average radius, arbitrary radius and the thickness of the cylinder which are clarified in Figure 1e. This last one is computed as follows [36,37,38,39]

(1) $$V_{CNT}^{*}=\frac{W_{CNT}}{W_{CNT}+\left(\frac{\rho_{CNT}}{\rho_{m}}\right)-\left(\frac{\rho_{CNT}}{\rho_{m}}\right)W_{CNT}}$$

where $W_{CNT}$ is the mass fraction of the CNTs, while $\rho_{CNT}$ and $\rho_{m}$ refer to the density of the CNTs and matrix, respectively. In Table 1, all of the patterns are defined in terms of the same total volume fraction $V_{CNT}^{*}$. In a FG-X distribution of CNTs, the top and bottom surfaces reach the maximum values, whereas the mid-plane is completely free of CNTs, with a linear increase of CNTs along the thickness. The contrary occurs for a FG-O distribution of CNTs, where the external surfaces are free of CNTs, the mid-surface of the plate is enriched with CNTs and a linear decrease of CNTs is established from the mid-plane to the external surfaces. In a FG-V distribution, the top surface is enriched of CNTs, the bottom one is completely free of CNTs, with a linear increase in the amount of CNTs from the bottom to the top surfaces. In a UD type, the volume fraction of CNTs maintains constant along the thickness. Figure 1 illustrates the whole distributions of $V_{CNT}$ as a function of the thickness coordinate.

The rule of mixture is here applied to define the effective material properties, namely, the Young’s moduli $E_{11}$, $E_{22}$, the shear modulus $G_{12}$, the density ρ, and the Poisson’s ratio $\nu_{12}$, as follows [40,41]:

(2) $$\left\{ \begin{array}{c} E_{11}=\eta_{1}V_{CNT}E_{11}^{CNT}+V_{m}E^{m}\\ \frac{\eta_{2}}{E_{22}}=\frac{V_{CNT}}{E_{22}^{CNT}}+\frac{V_{m}}{E^{m}}\;\\ \frac{\eta_{3}}{G_{12}}=\frac{V_{CNT}}{G_{12}^{CNT}}+\frac{V_{m}}{G^{m}}\\ \rho =V_{CNT}\rho^{CNT}+V_{m}\rho^{m}\\ \nu_{12}=V_{CNT}\nu_{12}^{CNT}+V_{m}\nu^{m} \end{array}\right.$$

where $\eta_{1}$, $\eta_{2}$ and $\eta_{3}$ refer to the efficiency parameters explaining the scale-dependent material properties, $V_{CNT}$ and $V_{m}$ are the volume fractions of the CNT and the matrix, respectively, which are related to each other as:

(3) $$V_{CNT}+V_{m}=1$$

The other effective mechanical properties $E_{33}$, $G_{13}$, $\nu_{31}$, and $\nu_{32}$, are described here below:

(4) $$E_{33}=E_{22}\;,\;G_{13}=G_{12}\;,\;\nu_{31}=\nu_{21}\;,\;\nu_{32}=\nu_{21}$$

### 2.2. Basic Equations

**Figure 2:**
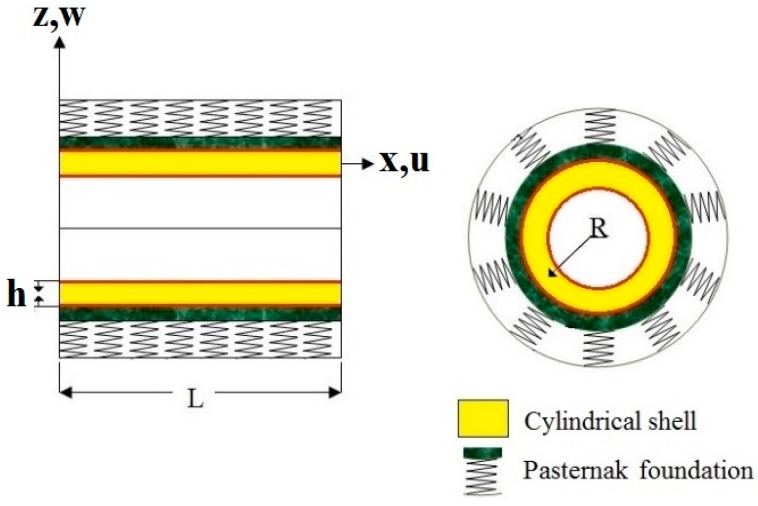
Scheme of the cylindrical pressure vessel surrounded by the Pasternak foundation.

According to the TSDT, the displacement field (see Figure 2) of the cylinder reads [42]:

(5) $$\left\{\begin{array}{c} u_{x}\\ u_{z} \end{array}\right\}=\left\{\begin{array}{c} u_{0}\left(x\right)\\ w_{0}\left(x\right) \end{array}\right\}+z\left\{\begin{array}{c} u_{1}\left(x\right)\\ w_{1}\left(x\right) \end{array}\right\}+z^{2}\left\{\begin{array}{c} u_{2}\left(x\right)\\ w_{2}\left(x\right) \end{array}\right\}+z^{3}\left\{\begin{array}{c} u_{3}\left(x\right)\\ w_{3}\left(x\right) \end{array}\right\}$$

where $u_{x}$ and $u_{z}$ are the axial and radial displacement components, respectively, and $u_{i}\;\left\{i=1,\;2,\;3\right\}$ and $w_{i}\left\{i=1,\;2,\;3\right\}$ are functions of x. Thus, the strain field can be obtained by derivation, together with the axial component $\varepsilon_{x}$, radial component $\varepsilon_{z}$, circumferential component $\varepsilon_{t}$ and shear component $\gamma_{xz}$, namely:

(6) $$\left\{ \begin{array}{c} \varepsilon_{x}=\frac{\partial u_{x}}{\partial x}=\frac{\partial u_{0}}{\partial x}+z\frac{\partial u_{1}}{\partial x}+z^{2}\frac{\partial u_{2}}{\partial x}+z^{3}\frac{\partial u_{3}}{\partial x}\\ \varepsilon_{z}=\frac{\partial u_{z}}{\partial z}=w_{1}+2zw_{2}+3z^{2}w_{3}\\ \varepsilon_{t}=\frac{u_{z}}{r}=\frac{w_{0}+zw_{1}+z^{2}w_{2}+z^{3}w_{3}}{R+z}\\ \gamma_{xz}=2\varepsilon_{xz}=\frac{\partial u_{x}}{\partial z}+\frac{\partial u_{z}}{\partial x}=u_{1}+2zu_{2}+\\ +3z^{2}u_{3}+\frac{\partial w_{0}}{\partial x}+z\frac{\partial w_{1}}{\partial x}+z^{2}\frac{\partial w_{2}}{\partial x}+z^{3}\frac{\partial w_{3}}{\partial x} \end{array}\right.$$

The stress-strain relations read as follows:

(7) $$\left[\begin{array}{c} \sigma_{x}\\ \sigma_{t}\\ \sigma_{z}\\ \tau_{xz} \end{array}\right]=\left[\begin{array}{cccc} Q_{11} & Q_{12} & Q_{13} & 0\\ Q_{21} & Q_{22} & Q_{23} & 0\\ Q_{31} & Q_{32} & Q_{33} & 0\\ 0 & 0 & 0 & Q_{55} \end{array}\right]\left[\begin{array}{c} \varepsilon_{x}-\alpha_{11}T\\ \varepsilon_{t}-\alpha_{22}T\\ \varepsilon_{z}-\alpha_{33}T\\ \gamma_{xz} \end{array}\right]$$

where,

(8) $$\begin{array}{c} Q_{11}=\frac{E_{11}}{\Delta }\left(1-\nu_{23}\nu_{32}\right),\;Q_{22}=\frac{E_{22}}{\Delta }\left(1-\nu_{13}\nu_{31}\right),\\ Q_{33}=\frac{E_{33}}{\Delta }\left(1-\nu_{21}\nu_{12}\right),Q_{55}=G_{13},\\ Q_{12}=\frac{E_{11}}{\Delta }\left(\nu_{21}+\nu_{31}\nu_{23}\right),\\ Q_{13}=\frac{E_{11}}{\Delta }\left(\nu_{31}+\nu_{21}\nu_{32}\right)\\ Q_{23}=\frac{E_{22}}{\Delta }\left(\nu_{32}+\nu_{12}\nu_{31}\right)\\ \Delta =1-\nu_{12}\nu_{21}-\nu_{23}\nu_{32}-\nu_{31}\nu_{13}-2\nu_{12}\nu_{32}\nu_{13} \end{array}$$

In Equation (7), T represents the increment in temperature from the reference state, and $\alpha_{11}$, $\alpha_{22}$ and $\alpha_{33}$ are the coefficients of thermal expansion.

On the other hand, the following constitutive relations must be considered, relating forces and displacements, due to the presence of the Pasternak foundation:

(9) $$F_{f}=k_{1}\left(u_{z=\frac{h}{2}}\right)-k_{2}\frac{\partial^{2}}{\partial x^{2}}\left(u_{z=\frac{h}{2}}\right)$$

$k_{1}$ and $k_{2}$ being the Pasternak coefficients. In Equation (9), the two kinematic terms can be expressed in the following expanded form:

(10) $$\left\{ \begin{array}{c} u_{z=\frac{h}{2}}=w_{0}+\frac{h}{2}w_{1}+{\left(\frac{h}{2}\right)}^{2}w_{2}+{\left(\frac{h}{2}\right)}^{3}w_{3}\\ \frac{\partial^{2}}{\partial x^{2}}\left(u_{z=\frac{h}{2}}\right)=\frac{\partial^{2}w_{0}}{\partial x^{2}}+\frac{h}{2}\frac{\partial^{2}w_{1}}{\partial x^{2}}+{\left(\frac{h}{2}\right)}^{2}\frac{\partial^{2}w_{2}}{\partial x^{2}}+{\left(\frac{h}{2}\right)}^{3}\frac{\partial^{2}w_{3}}{\partial x^{2}} \end{array}\right.$$

Substitution of Equation (10) into Equation (9) yields the following expression:

(11) $$F_{f}=k_{1}\left(w_{0}+\frac{h}{2}w_{1}+{\left(\frac{h}{2}\right)}^{2}w_{2}+{\left(\frac{h}{2}\right)}^{3}w_{3}\right)-k_{2}\left[\frac{\partial^{2}w_{0}}{\partial x^{2}}+\frac{h}{2}\frac{\partial^{2}w_{1}}{\partial x^{2}}+{\left(\frac{h}{2}\right)}^{2}\frac{\partial^{2}w_{2}}{\partial x^{2}}+{\left(\frac{h}{2}\right)}^{3}\frac{\partial^{2}w_{3}}{\partial x^{2}}\right]$$

Therefore, we can define the variation of the strain energy as follows:

(12) $$\delta U=\int \sigma_{ij}\delta \varepsilon_{ij}d\forall =\int \left[\sigma_{x}\delta \varepsilon_{x}+\sigma_{t}\delta \varepsilon_{t}+\sigma_{z}\delta \varepsilon_{z}+\tau_{xz}\delta \gamma_{xz}\right]d\forall$$

By the substitution of Equation (6) into Equation (12), Equation (12) can be rewritten as:

(13) $$\begin{array}{c} \delta U=\iint (\sigma_{x}\left\{\frac{\partial \delta u_{0}}{\partial x}+z\frac{\partial \delta u_{1}}{\partial x}+z^{2}\frac{\partial \delta u_{2}}{\partial x}+z^{3}\frac{\partial \delta u_{3}}{\partial x}\right\}+\sigma_{t}\left\{\frac{1}{R+z}\left[\delta w_{0}+z\delta w_{1}+z^{2}\delta w_{2}+z^{3}\delta w_{3}\right]\right\}\\ \;+\sigma_{z}\left\{\delta w_{1}+2z\delta w_{2}+3z^{2}\delta w_{3}\right\}\\ \;+\tau_{xz}\left\{\delta u_{1}+2z\delta u_{2}+3z^{2}\delta u_{3}+\frac{\partial \delta w_{0}}{\partial x}+z\frac{\partial \delta w_{1}}{\partial x}+z^{2}\frac{\partial \delta w_{2}}{\partial x}+z^{3}\frac{\partial \delta w_{3}}{\partial x}\right\})2\pi (R+z)dzdx \end{array}$$

For simplification purposes, Equation (13) can be rewritten in terms of internal stress resultants as follows:

(14) $$\begin{array}{c} \delta U=2\pi \int [N_{x}\frac{\partial \delta u_{0}}{\partial x}+M_{x}\frac{\partial \delta u_{1}}{\partial x}+P_{x}\frac{\partial \delta u_{2}}{\partial x}+S_{x}\frac{\partial \delta u_{3}}{\partial x}+N_{t}\delta w_{0}+M_{t}\delta w_{1}+P_{t}\delta w_{2}+S_{t}\delta w_{3}+N_{z}\delta w_{1}+M_{z}\delta w_{2}+\\ P_{z}\delta w_{3}+Q_{x}\delta u_{1}+Q_{x}\frac{\partial \delta w_{0}}{\partial x}+2M_{xz}\delta u_{2}+M_{xz}\frac{\partial \delta w_{1}}{\partial x}+3P_{xz}\delta u_{3}+P_{xz}\frac{\partial \delta w_{2}}{\partial x}+S_{xz}\frac{\partial \delta w_{3}}{\partial x}]dx \end{array}$$

where the internal stress resultants can be introduced as follows:

(15) $$\begin{array}{c} N_{x}=\int \sigma_{x}\left(R+z\right)dz\\ P_{x}=\int \sigma_{x}z^{2}\left(R+z\right)dz\\ N_{t}=\int \sigma_{t}dz\\ P_{t}=\int \sigma_{t}z^{2}dz\\ N_{z}=\int \sigma_{z}\left(R+z\right)dz\\ P_{z}=3\int \sigma_{z}z^{2}\left(R+z\right)dz\\ M_{xz}=\int \tau_{xz}z\left(R+z\right)dz\\ S_{xz}=\int \tau_{xz}z^{3}\left(R+z\right)dz\\ M_{x}=\int \sigma_{x}z\left(R+z\right)dz\\ S_{x}=\int \sigma_{x}z^{3}\left(R+z\right)dz\\ M_{t}=\int \sigma_{t}zdz\\ S_{t}=\int \sigma_{t}z^{3}dz\\ M_{z}=2\int \sigma_{z}z\left(R+z\right)dz\\ Q_{x}=\int \tau_{xz}\left(R+z\right)dz\\ P_{xz}=\int \tau_{xz}z^{2}\left(R+z\right)dz \end{array}$$

In addition, we can define the variation of the external work as follows:

(16) $$\delta W=\int \left[P_{i}\delta w_{z}{|}_{z=-\frac{h}{2}}-F_{f}\delta w_{z}{|}_{z=\frac{h}{2}}\right]dA$$

where $P_{i}$ is the internal pressure, here set to $P_{i}=70\;\mathrm{MPa}.$

By partial integration of Equation (14) and by substitution of Equations (6) and (11) into Equation (16), get the following variations of the strain energy and energy of the external work:

(17) $$\delta U=2\pi \int \left[\begin{array}{c} -\frac{\partial N_{x}}{\partial x}\delta u_{0}+\left(-\frac{\partial M_{x}}{\partial x}+Q_{x}\right)\delta u_{1}\\ +\left(-\frac{\partial P_{x}}{\partial x}+2M_{xz}\right)\delta u_{2}+\left(-\frac{\partial S_{x}}{\partial x}+3P_{xz}\right)\delta u_{3}\\ +\left(N_{t}-\frac{\partial Q_{x}}{\partial x}\right)\delta w_{0}+\left(M_{t}+N_{z}-\frac{\partial M_{xz}}{\partial x}\right)\delta w_{1}\\ +\left(P_{t}+M_{z}-\frac{\partial P_{xz}}{\partial x}\right)\delta w_{2}+\left(S_{t}+P_{z}-\frac{\partial S_{xz}}{\partial x}\right)\delta w_{3} \end{array}\right]dx$$

(18) $$\delta W=2\pi \int \left[\begin{array}{c} \left\{P_{i}\left(R-\frac{h}{2}\right)-F_{f}\left(R+\frac{h}{2}\right)\right\}\delta w_{0}\\ +\left\{-P_{i}\left(R-\frac{h}{2}\right)\frac{h}{2}-F_{f}\left(R+\frac{h}{2}\right)\frac{h}{2}\right\}\delta w_{1}\\ +\left\{P_{i}\left(R-\frac{h}{2}\right){\left(\frac{h}{2}\right)}^{2}-F_{f}\left(R-\frac{h}{2}\right){\left(\frac{h}{2}\right)}^{2}\right\}\delta w_{2}\\ +\left\{P_{i}\left(R-\frac{h}{2}\right){\left(\frac{h}{2}\right)}^{3}-F_{f}\left(R-\frac{h}{2}\right){\left(\frac{h}{2}\right)}^{3}\right\}\delta w_{3} \end{array}\right]dx$$

The application of the Hamilton’s principle gives the following governing equations of the problem:

(19) δΠ=δU−δW=0

and the substitution of Equations (17) and (18) into Equation (19) gives the following differential equations in terms of internal stress resultants:

(20) $$\left\{ \begin{array}{c} \delta u_{0}\;:\;-\frac{\partial N_{x}}{\partial x}=0\\ \delta u_{1}\;:\;-\frac{\partial M_{x}}{\partial x}+Q_{x}=0\\ \delta u_{2}\;:\;-\frac{\partial P_{x}}{\partial x}+2M_{xz}=0\\ \delta u_{3}\;:\;-\frac{\partial S_{x}}{\partial x}+3P_{xz}=0\\ \delta w_{0}\;:N_{t}-\frac{\partial Q_{x}}{\partial x}-\left(P_{i}\left(R-\frac{h}{2}\right)-F_{f}\left(R+\frac{h}{2}\right)\right)=0\\ w_{1}\;:\;M_{t}+N_{z}-\frac{\partial M_{xz}}{\partial x}+\left\{P_{i}\left(R-\frac{h}{2}\right)\frac{h}{2}+F_{f}\left(R+\frac{h}{2}\right)\frac{h}{2}\right\}=0\;\\ \delta w_{2}\;:\;P_{t}+M_{z}-\frac{\partial P_{xz}}{\partial x}-\left\{P_{i}\left(R-\frac{h}{2}\right){\left(\frac{h}{2}\right)}^{2}-F_{f}\left(R-\frac{h}{2}\right){\left(\frac{h}{2}\right)}^{2}\right\}=0\\ \delta w_{3}\;:\;S_{t}+P_{z}-\frac{\partial S_{xz}}{\partial x}-\left\{P_{i}\left(R-\frac{h}{2}\right){\left(\frac{h}{2}\right)}^{3}-F_{f}\left(R-\frac{h}{2}\right){\left(\frac{h}{2}\right)}^{3}\right\}=0 \end{array}\right.$$

By substitution of Equations (15) and (11) into Equation (20), we obtain the following set of equations in the matrix form:

(21) $$G_{1}X^{\prime \prime }+G_{2}X^{\prime }+G_{3}X=F$$

where the displacement vector X, the coefficient matrixes ($G_{1}$,$\;G_{2}$ and $G_{3}$) and the force vector F are defined as follows:

(22) $$X={\left\{u_{0}\;u_{1}\;u_{2}\;u_{3}\;w_{0}\;w_{1}\;w_{2}\;w_{3}\right\}}^{T}$$

(23) $$G_{1}=[\begin{array}{cccccccc} -A_{1} & -A_{2} & -A_{3} & -A_{4} & 0 & 0 & 0 & 0\\ -A_{2} & -A_{3} & -A_{4} & -A_{5} & 0 & 0 & 0 & 0\\ -A_{3} & -A_{4} & -A_{5} & -A_{6} & 0 & 0 & 0 & 0\\ -A_{4} & -A_{5} & -A_{6} & -A_{7} & 0 & 0 & 0 & 0\\ 0 & 0 & 0 & 0 & -A_{8}-B_{1} & -A_{9}-\frac{B_{1}h}{2} & -A_{10}-\frac{B_{1}h^{2}}{4} & -A_{11}-\frac{B_{1}h^{3}}{8}\\ 0 & 0 & 0 & 0 & -A_{9}-\frac{B_{1}h}{2} & -A_{10}-\frac{B_{1}h^{2}}{4} & -A_{11}-\frac{B_{1}h^{3}}{8} & -A_{12}-\frac{B_{1}h^{4}}{16}\\ 0 & 0 & 0 & 0 & -A_{10}-\frac{B_{2}h^{2}}{4} & -A_{11}-\frac{B_{2}h^{3}}{8} & -A_{12}-\frac{B_{2}h^{4}}{16} & -A_{13}-\frac{B_{2}h^{5}}{32}\\ 0 & 0 & 0 & 0 & -A_{11}-\frac{B_{2}h^{3}}{8} & -A_{12}-\frac{B_{2}h^{4}}{16} & -A_{13}-\frac{B_{2}h^{5}}{32} & -A_{14}-\frac{B_{2}h^{6}}{64} \end{array}]$$

(24) $$G_{2}={\left[\begin{array}{cc} 0 & G_{2_{r}}\\ G_{2_{l}} & 0 \end{array}\right]}_{8\times 8}$$

(25) $$G_{2_{r}}=\left[\begin{array}{cccc} -A_{15} & -A_{16}-A_{22} & -A_{17}-2A_{23} & -A_{18}-3A_{24}\\ -A_{16}+A_{8} & -A_{17}-A_{23}+A_{9} & A_{10}-A_{18}-2A_{24} & A_{11}-A_{19}-3A_{25}\\ -A_{17}+2A_{9} & 2A_{10}-A_{18}-A_{24} & 2A_{11}-A_{19}-2A_{25} & 2A_{12}-A_{20}-3A_{26}\\ 3A_{10}-A_{18} & 3A_{11}-A_{19}-A_{25} & 3A_{12}-A_{20}-2A_{26} & 3A_{13}-A_{21}-3A_{27} \end{array}\right]$$

(26) $$G_{2_{l}}=\left[\begin{array}{cccc} A_{15} & A_{16}-A_{8} & A_{17}-2A_{9} & -3A_{10}+A_{18}\\ A_{16}+A_{22} & A_{17}+A_{23}-A_{9} & -2A_{10}+A_{18}+A_{24} & -3A_{11}+A_{19}+A_{25}\\ A_{17}+2A_{23} & -A_{10}+A_{18}+2A_{24} & -2A_{11}+A_{19}+2A_{25} & -3A_{12}+A_{20}+2A_{26}\\ A_{18}+3A_{24} & -A_{11}+A_{19}+3A_{25} & -2A_{12}+A_{20}+3A_{26} & -3A_{13}+A_{21}+3A_{27} \end{array}\right]$$

(27) $$G_{3}={\left[\begin{array}{cc} G_{3_{l}} & 0\\ 0 & G_{3_{r}} \end{array}\right]}_{8\times 8}$$

(28) $$G_{3_{r}}=\left[\begin{array}{cccc} A_{28}+B_{3} & A_{29}+A_{35}+\frac{B_{3}h}{2} & A_{30}+2A_{36}+\frac{B_{3}h^{2}}{4} & A_{31}+3A_{37}+\frac{B_{3}h^{3}}{8}\\ A_{29}+A_{35}+\frac{B_{3}h}{2} & A_{30}+2A_{36}+A_{41}+\frac{B_{3}h^{2}}{4} & A_{31}+3A_{37}+2A_{42}+\frac{B_{3}h^{3}}{8} & A_{32}+4A_{38}+3A_{43}+\frac{B_{3}h^{4}}{16}\\ A_{30}+2A_{36}+\frac{B_{4}h^{2}}{4} & A_{31}+3A_{37}+2A_{42}+\frac{B_{4}h^{3}}{8} & A_{32}+4A_{38}+4A_{43}+\frac{B_{4}h^{4}}{16} & A_{33}+5A_{39}+6A_{44}+\frac{B_{4}h^{5}}{32}\\ A_{31}+3A_{37}+\frac{B_{4}h^{3}}{8} & A_{32}+4A_{38}+3A_{43}+\frac{B_{4}h^{4}}{16} & A_{33}+5A_{39}+6A_{44}+\frac{B_{4}h^{5}}{32} & A_{34}+6A_{40}+9A_{45}+\frac{B_{4}h^{6}}{64} \end{array}\right]$$

(29) $$G_{3_{l}}=\left[\begin{array}{cccc} 0 & 0 & 0 & 0\\ 0 & A_{8} & 2A_{9} & 3A_{10}\\ 0 & 2A_{9} & 4A_{10} & 6A_{11}\\ 0 & 3A_{10} & 6A_{11} & 9A_{12} \end{array}\right]$$

(30) $$F=\left[\begin{array}{c} 0\\ 0\\ 0\\ 0\\ A_{46}-B_{5}\\ A_{47}+A_{50}+\frac{B_{5}h}{2}\\ A_{48}+2A_{51}-\frac{B_{5}h^{2}}{4}\\ A_{49}+3A_{52}-\frac{B_{5}h^{3}}{8} \end{array}\right]$$

Additional details about the analytical expression of the coefficients $A_{i}\;\left\{i=1\ldots 52\right\}$ and $B_{i}\left\{i=1\ldots 5\right\}$, can be found in Appendix A.

## 3. Solution Procedure

The problem above is solved including both homogeneous and particular solutions. Here below the complete solving procedure, that embraces the following 4 steps

Step 1. The particular solution of Equation (18) is obtained as [43,44,45]:

(31) $$G_{3}X_{p}=F\to X_{p}={G_{3}}^{-1}F$$

Step 2. The eigenvalues $m_{i}\;\left\{i=1,\;2,\;3\right\}$ are obtained by solving the characteristic equation:

(32) $$G_{3}X_{p}=F\to X_{p}={G_{3}}^{-1}F$$

Step 3. The associated eigenvectors $v_{i}\left\{i=1,\;2,\;3\right\}$ are determined as:

(33) $$(G_{1}m^{2}+G_{2}m+G_{3})v=0$$

Step 4. The displacement components are computed by substitution of the eigenvalues $m_{i}$ and eigenvectors $v_{i}\;$ into the following equation:

(34) $$X^{j}=X_{h}^{j}+X_{p}=\overset{16}{\underset{i=1}{\sum }}c_{i}v_{j}^{i}e^{m_{i}x}+X_{p}\;\left(j=1\ldots 8\right)$$

where $c_{i}$ refers to the unknown coefficients determined by enforcing the appropriate boundary conditions, here assumed as clamped-clamped at each side, namely:

(35) $$at\;x=0\;and\;x=l\to \{\begin{array}{c} u_{0}=u_{1}=u_{2}=u_{3}=0\\ w_{0}=w_{1}=w_{2}=w_{3}=0 \end{array}$$

## 4. Validation

**Figure 3:**
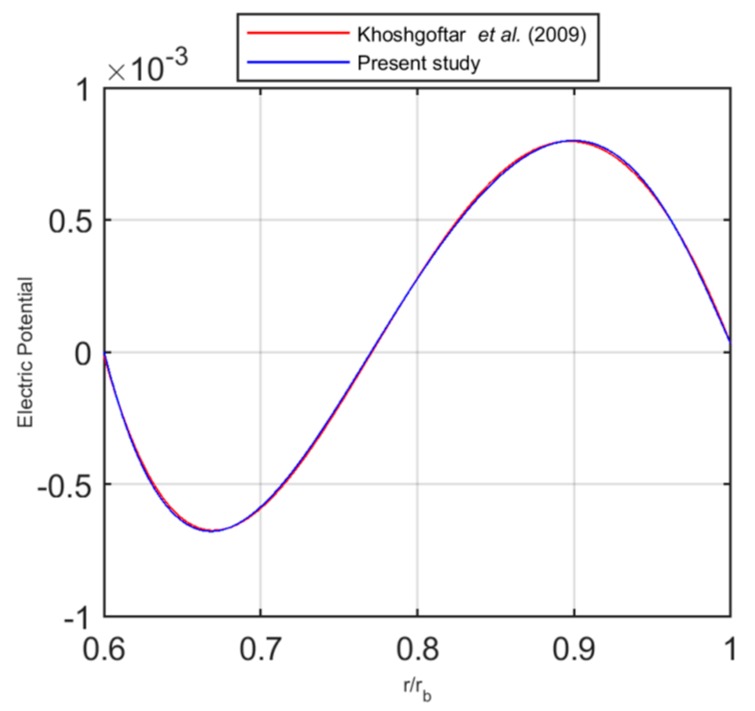
Distribution of the electric potential along the thickness.

Khoshgoftar et al. [21,45] studied the thermoelastic behaviour of a FG piezoelectric cylindrical structure, whose results have been here selected for validation purposes. In detail, the accuracy of the solution procedure is evaluated for l=0, where l is the non-homogeneous parameter. A comparative evaluation of the results is shown in Figure 3 in terms of distribution of the electrical potential along the thickness for a thick walled cylinder made of piezoelectric material. The perfect agreement between our results and predictions from the literature confirms the reliability and accuracy of the proposed formulation.

## 5. Numerical Results

We illustrate the use of the proposed formulation, here applied for a cylinder with inner radius $r_{i}=0.04\;m$, outer radius $r_{o}=0.05\;m$ and length L=0.5 m. The structure is made of a polyethylmethacrylate (PMMA) material with the same mechanical properties for the matrix as found in Kiani [22], i.e., $E_{m}=2.5\;\mathrm{GPa}$, $\alpha_{m}=45\times 10^{-6}\frac{1}{K}$, $\nu_{m}=0.34$. A SWCNT is here considered as fiber reinforcement, with geometrical, mechanical and thermal properties, as detailed in Table 2.

As a comparison, we assume three different volume fractions of CNTs, as follows [22]:

$$\begin{array}{c} \eta_{1}=0.137,\;\eta_{2}=1.022,\;\eta_{3}=0.7\eta_{2}\;\mathrm{for}\;V_{CNT}^{*}=0.12\\ \eta_{1}=0.142,\;\eta_{2}=1.626,\;\eta_{3}=0.7\eta_{2}\;\mathrm{for}\;V_{CNT}^{*}=0.17\\ \eta_{1}=0.141,\;\eta_{2}=1.585,\;\eta_{3}=0.7\eta_{2}\;\mathrm{for}\;V_{CNT}^{*}=0.28 \end{array}$$

Additionally, we consider different CNT distributions and different Pasternak coefficients for comparative purposes, while applying both the FSDT and TSDT to investigate the kinematic and static response of the reinforced structure.

### 5.1. Kinematic Response

**Figure 4:**
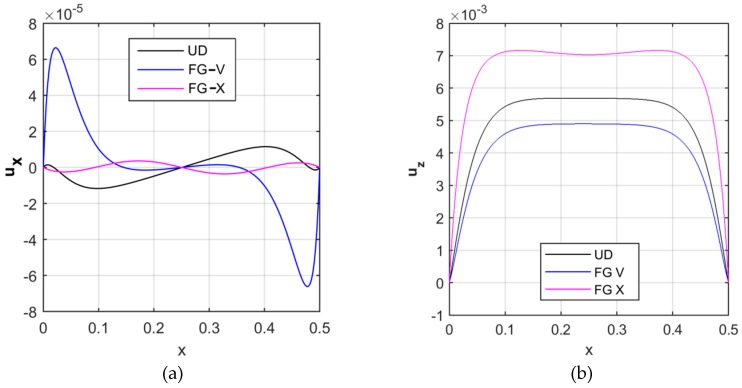
Longitudinal distribution of the axial (**a**) and radial (**b**) displacement components [m], for different reinforcement patterns.

First, we analyze the effect of the reinforcement distribution on the kinematic response of the structure. Figure 4 shows the longitudinal distribution of the axial displacement $u_{x}$ (Figure 4a) and radial displacement $u_{z}$ (Figure 4b) at the middle surface (z=0), for a UD, FG-X and FG-V pattern, as well as for a fixed volume fraction $V_{CNT}^{*}=0.12$ and a null value of the Pasternak coefficients $k_{1}=k_{2}=0$.

Based on the numerical results, the boundary conditions are clearly satisfied at two ending supports of the cylindrical pressure vessel. In addition, it is worth noting that the maximum radial displacement is reached under a FG-X distribution, whereas the maximum axial displacement is obtained for a FG-V distribution.

**Figure 5:**
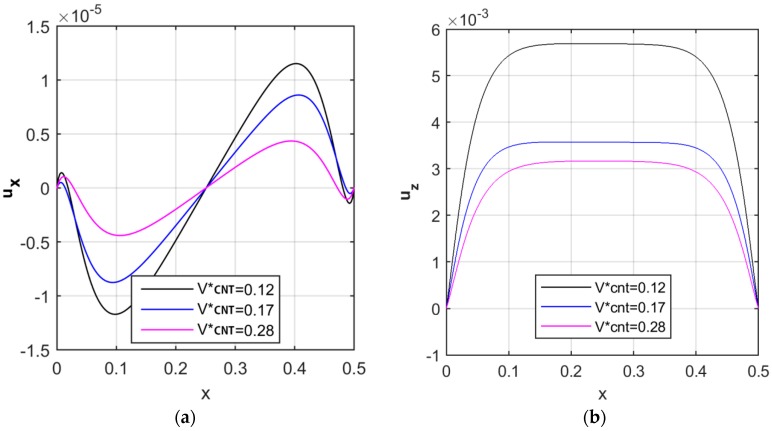
Longitudinal distribution of the axial (**a**) and radial (**b**) displacement components [m], for different volume fractions.

A further check is evaluated on the sensitivity of the structural response to the volume fraction of reinforcement. Figure 5 illustrates the variation of the axial and radial displacements at the middle surface (i.e., for z=0) along the longitudinal direction, by applying different volume fractions of FG-CNTRC, and considering a UD reinforcement pattern. Also in this case, we keep the Pasternak coefficients $k_{1},\;k_{2}$ equal to zero. A significant decrease in both axial and radial displacements is noticed for increasing volume fractions of CNTs, in line with findings by Arefi et al. [6]. An increased volume fraction of CNTs, indeed, yields to an increased structural stiffness, with a consistent reduction of the kinematic quantities. The variations of the longitudinal and radial displacements along the radial direction are listed in Table 3 and Table 4 at various longitudinal positions. It is worth noticing that the maximum radial displacements are reached at the middle surface of the cylindrical shell. In addition, the maximum longitudinal displacements occur near one quarter of the cylindrical shell.

**Figure 6:**
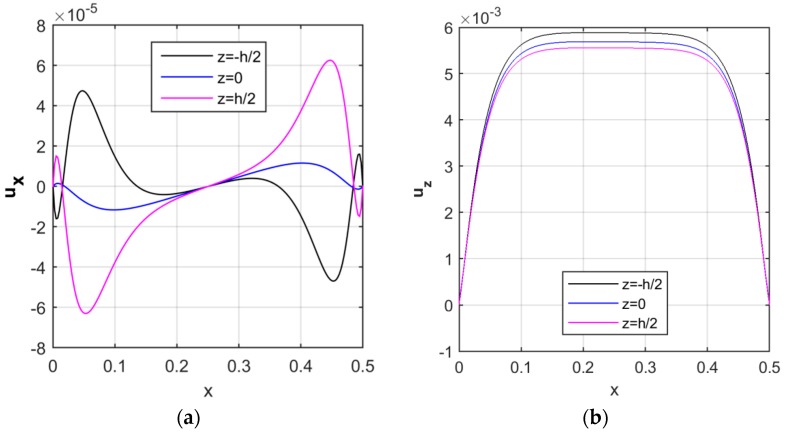
Longitudinal distribution of the axial (**a**) and radial (**b**) displacement components [m], in the middle and external surfaces.

Due to the application of the TSDT, the displacement components are expected to vary along the radial direction, as represented in Figure 6 for a volume fraction $V_{CNT}^{*}=0.12$, a uniform distribution of CNTs, and null values of the Pasternak coefficients $k_{1}=k_{2}=0$. As visible in Figure 6a, the axial displacement assumes an asymmetric behavior at each surface along the longitudinal direction, while reaching the minimum value at the mid-surface (i.e., for z=0), and the maximum values at the external lateral surfaces (i.e., for $z=\frac{-h}{2}$ and $z=\frac{h}{2}$).

In addition, moving from the inner surface $\left(z=\frac{-h}{2}\right)$ to the outer one ($z=\frac{h}{2})$ of the cylinder, the radial displacement reduces slightly, and attains the maximum values at the mid length of the structure (see Figure 6b).

**Figure 7:**
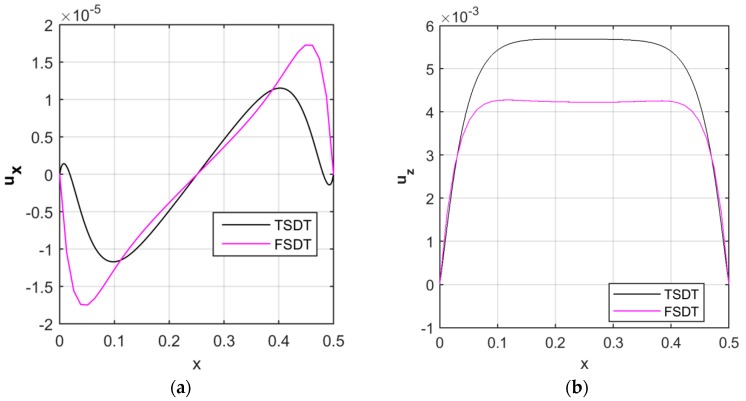
Longitudinal distribution of the axial (**a**) and radial (**b**) displacement components [m], according to the third-order shear deformation theory (TSDT) and first-order shear deformation theory (FSDT).

The efficiency of the proposed TSDT formulation is also verified against the FSDT, through a comparative evaluation of the kinematic results in the axial and radial direction. As expected, more accurate results can be obtained by applying a TSDT compared to the other based on lower order theories [33], as clearly shown in Figure 7. In more detail, the TSDT–based axial displacement in the longitudinal direction is lower than the FSDT estimate (see Figure 7a), whereas the TSDT-based radial displacement is always higher than the FSDT prediction along the whole specimen (see Figure 7b).

### 5.2. Tensional Response

**Figure 8:**
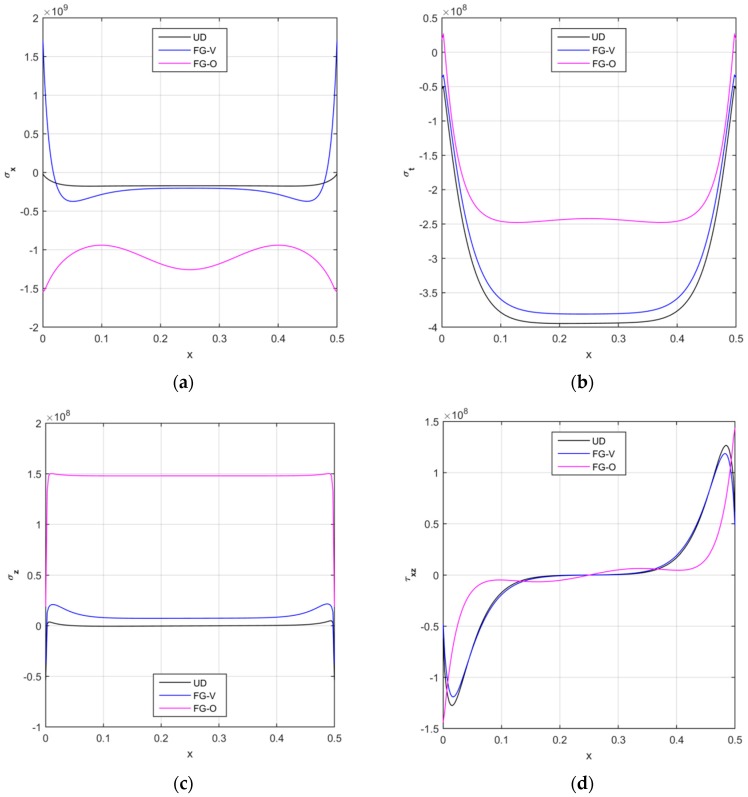
The longitudinal distribution of the axial (**a**), circumferential (**b**), radial (**c**), and shear (**d**) stress components [Pa] at the mid-surface of the CNTRC cylinder for different reinforcement distributions.

**Figure 9:**
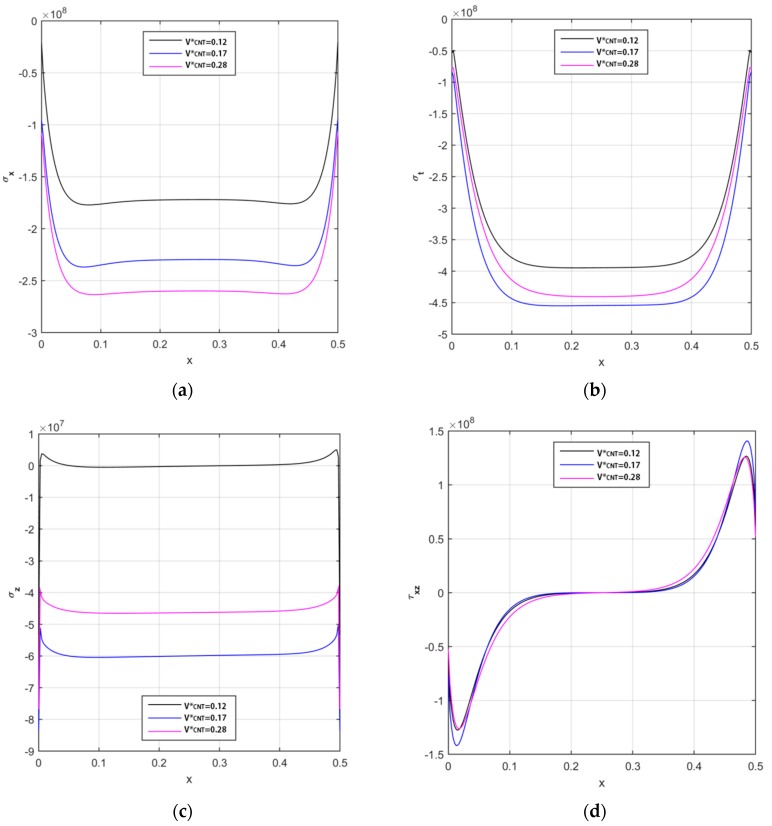
The longitudinal distribution of the axial (**a**), circumferential (**b**), radial (**c**), and shear (**d**) stress components [Pa] of CNTRC cylinder for different volume fractions.

A similar parametric study is also repeated from a tensional point of view, in terms of axial, circumferential, radial and shear stresses along the longitudinal direction, and for different reinforcement patterns. Based on the numerical results in Figure 8, a FG-O pattern of CNTs seems to yield to the maximum values for the axial and radial stresses and to the minimum values for the circumferential and shear components except for the boundary zones. The stress response given by a UD and a FG-V distribution, instead, is almost similar for each component along the whole structure, with the highest numerical differences nearby the two clamped sides. The stress response can be affected significantly by the volume fraction of the reinforcement, whose variation is plotted in Figure 9, assuming volume fractions $V_{CNT}^{*}=0.12,\;0.17,\;0.28$. This parametric investigation is here tackled for a UD pattern, but could be similarly repeated for all the other reinforcement distributions. Based on the numerical results, a general increase in magnitude is observed for the axial, circumferential, and radial stress components, and for a volume fraction V^*^_CNT_ higher than 0.12 (see Figure 9a–c).

Only the shear stress seems to be almost insensitive to the volume fraction (see Figure 9d), in agreement with findings by Arefi et al. [6].

**Figure 10:**
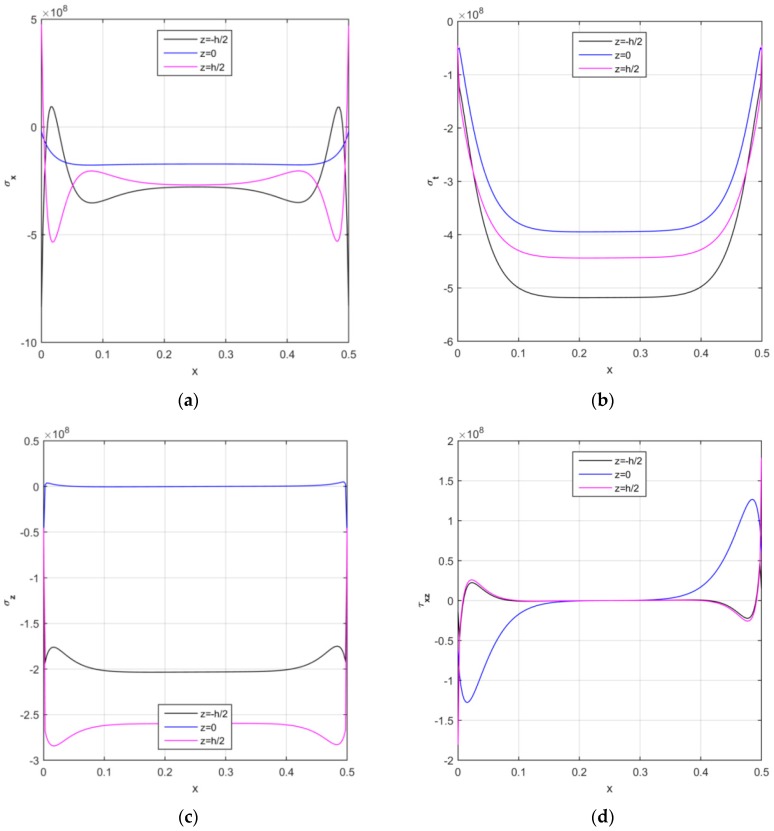
The longitudinal distribution of the axial (**a**), circumferential (**b**), radial (**c**), and shear (**d**) stress components [Pa] at different surfaces of the CNTRC cylinder.

In addition, Figure 10 represents the longitudinal distribution of the stress components for the middle and external surfaces of the structure (i.e., for $z=\frac{-h}{2},\;z=0,\;z=\frac{h}{2}$, respectively). It is worth observing that the TSDT allows all stress components to assume different magnitudes through the thickness direction, as can be seen in Figure 10a–d, for $V_{CNT}^{*}=0.12,\;FG-UD,\;k_{1}=k_{2}=0$.

**Figure 11:**
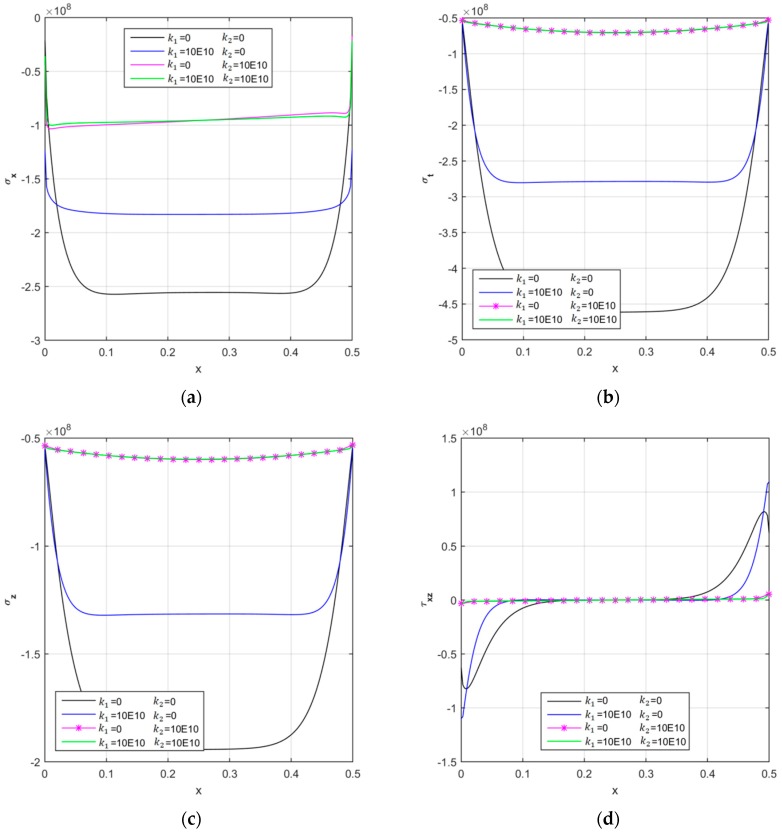
The longitudinal distribution of the axial (**a**), circumferential (**b**), radial (**c**), and shear (**d**) stress components [Pa] of the CNTRC cylinder for different Pasternak coefficients.

The last parametric study focuses on the sensitivity of the stress response to the Pasternak parameters of the elastic foundation. Figure 11 plots the main curves for different combinations of $k_{1}$ and $k_{2}$, while keeping $V_{CNT}^{*}=0.12,\;FG-UD,\;z=0$. The numerical results in Figure 11 clearly state that lower magnitudes can be obtained for each stress component by increasing both Pasternak parameters. This is strictly related to a general increase in stiffness of the foundation for increasing values of $k_{1}$ and $k_{2}$, with an expected sensitive reduction of the kinematic quantities.

### 5.3. Radial Distribution of Results

**Figure 12:**
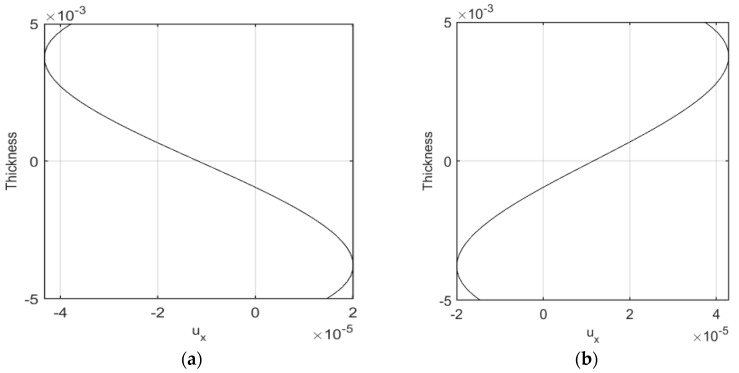
Maximum values of the axial deformation along the thickness direction at different longitudinal positions, at x = 0.1 (**a**) and x = 0.4 (**b**).

**Figure 13:**
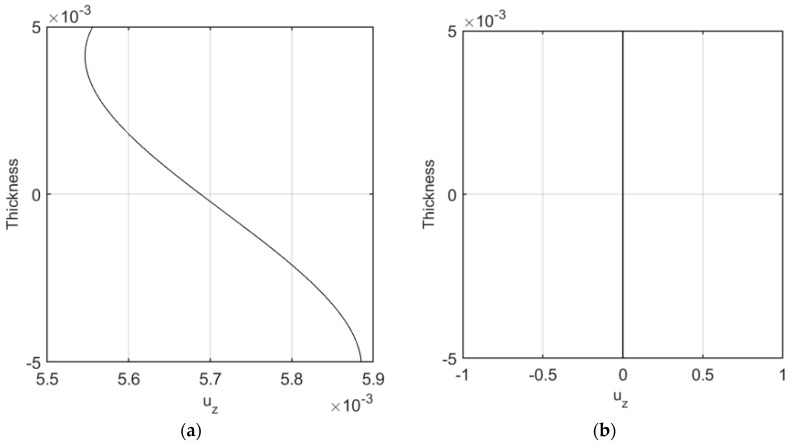
Maximum values of the radial deformation along the thickness direction at different longitudinal positions, at (**a**) x = 0.25 and (**b**) x = 0.5.

In this section, the radial distribution of numerical results are provided. For this purpose, the radial and axial deformation of the cylindrical shell are plotted along the radial direction at a defined longitudinal position. Figure 12a,b show the radial distribution of the longitudinal deformation at x = 0.1 and 0.4, respectively. One can conclude that the maximum longitudinal deformations occur at the external surfaces while the minimum values occur at the inner surfaces. Figure 13a,b plot the radial distribution of the radial deformation at x = 0.25 and 0.5, respectively. One can conclude that maximum radial deformations occur at the inner surfaces while the minimum deformations involve the outer surfaces.

## 6. Conclusions

The third-order shear deformation theory (TSDT) is employed in this work to study the thermo-elastic response of FG-CNTRC cylindrical pressure vessels resting on a Pasternak foundation. The rule of mixture is adopted herein for computing the effective material mechanical properties, whereby the governing equations of the problem are derived from Hamilton’s principle and solved as a classical eigenvalue problem. A parametric investigation aims at investigating the sensitivity of the static and kinematic response to some important parameters such as the reinforcement distribution, its volume fraction, or the Pasternak parameters related to the elastic foundation. Based on the numerical results, the main conclusions can be pointed out as:

- The accuracy of higher order theories, such as the TSDT, and lower order theories, such as the FSDT, must be determined comparatively with respect to the experimental results, but it is expected that a TSDT provides a more accurate structural response due to its capability to capture the variation of the static and kinematic responses through the thickness of the cylindrical structure.
- An increased volume fraction within the composite material yields to a reduction of the radial and axial displacement components, together with an increase of the stress field.
- An increasing stiffness of the foundation is reached for increasing values of the Pasternak parameters, with a consistent reduction of the displacement and stress field.
- Focusing on the reinforcement distributions, the maximum stress is obtained for a uniform distribution UD of CNTs, whereby the minimum stress is obtained for a FG-O distribution. At the same time, the minimum displacement is obtained for a FG-V distribution, while the maximum displacement is obtained for a FG-X pattern.

The conclusions above could be of interest for engineers and designers of mechanical and electronic devices, whose thermo-mechanical study requires an appropriate selection of the analytical and numerical tools, in order to ensure the feasibility of results.

## Figures and Tables

**Table 1:** Volume fraction of CNTs as a function of thickness coordinate for various cases of CNTs distribution [22].

CNTs Distribution	$V_{CNT}$
UD	$V_{CNT}=V_{CNT}^{*}$
FG-*X*	$V_{CNT}=4\left(\frac{\left|r-R\right|}{h}\right)V_{CNT}^{*}$
FG-*V*	$V_{CNT}=2\left(\frac{r-R}{h}+0.5\right)V_{CNT}^{*}$
FG-O	$V_{CNT}=\left(-4\frac{\left|r-R\right|}{h}+2\right)V_{CNT}^{*}$

**Table 2:** Thermo-mechanical properties of (10, 10) armchair single-walled carbon nanotube (SWCNT) (tube length = 9.26 nm, tube mean radius = 0.68 nm, tube thickness = 0.067 nm) [22].

T [K]	$E_{11}^{CNT}\;\left[TPa\right]$	$E_{22}^{CNT}\;\left[TPa\right]$	$G_{12}^{CNT\;}\left[TPa\right]$	$\nu_{12}^{CNT}$	$\alpha_{11}^{CNT}\;\left[10^{-6}\frac{1}{K}\right]$	$\alpha_{22}^{CNT\;}\left[10^{-6}\frac{1}{K}\right]$
300	5.6466	7.0800	1.9445	0.175	3.4584	5.1682

**Table 3:** Variation of the longitudinal displacement field along the radial direction at different longitudinal positions.

z [m]	$u_{x}\;\left[m\right]$				
	x=0	x=l/4	x=l/2	x=3l/4	x=l
−0.005	0	3.04 × 10^−6^	0	−3.12 × 10^−6^	0
−0.0044	0	4.3 × 10^−6^	0	−4.38 × 10^−6^	0
−0.0038	0	4.48 × 10^−6^	0	−4.55 × 10^−6^	0
−0.0032	0	3.71 × 10^−6^	0	−3.79 × 10^−6^	0
−0.0026	0	2.14 × 10^−6^	0	−2.23 × 10^−6^	0
−0.0021	0	−8.28 × 10^−8^	0	−2.66 × 10^−8^	0
−0.0015	0	−2.81 × 10^−6^	0	2.682 × 10^−6^	0
−0.0009	0	−5.91 × 10^−6^	0	5.748 × 10^−6^	0
−0.0003	0	−9.22 × 10^−6^	0	9.029 × 10^−6^	0
0.0003	0	−1.26 × 10^−5^	0	1.238 × 10^−5^	0
0.0009	0	−1.59 × 10^−5^	0	1.565 × 10^−5^	0
0.0015	0	−1.9 × 10^−5^	0	1.871 × 10^−5^	0
0.0021	0	−2.17 × 10^−5^	0	2.139 × 10^−5^	0
0.0026	0	−2.39 × 10^−5^	0	2.357 × 10^−5^	0
0.0032	0	−2.54 × 10^−5^	0	2.509 × 10^−5^	0
0.0038	0	−2.61 × 10^−5^	0	2.582 × 10^−5^	0
0.0044	0	−2.59 × 10^−5^	0	2.559 × 10^−5^	0
0.005	0	−2.46 × 10^−5^	0	2.428 × 10^−5^	0

**Table 4:** Variation of the radial displacement field along the radial direction at different longitudinal positions.

z [m]	$u_{z}\;\left[m\right]$				
	x=0	x=l/4	x=l/2	x=3l/4	x=l
−0.005	0	0.005791	0.005885	0.005767	0
−0.0044	0	0.005785	0.00588	0.005762	0
−0.0038	0	0.005773	0.005867	0.00575	0
−0.0032	0	0.005754	0.005848	0.005731	0
−0.0026	0	0.005731	0.005825	0.005708	0
−0.0021	0	0.005705	0.005798	0.005681	0
−0.0015	0	0.005675	0.005768	0.005652	0
−0.0009	0	0.005644	0.005736	0.005621	0
−0.0003	0	0.005612	0.005704	0.005589	0
0.0003	0	0.005581	0.005672	0.005557	0
0.0009	0	0.005551	0.005642	0.005527	0
0.0015	0	0.005523	0.005614	0.0055	0
0.0021	0	0.005499	0.005589	0.005476	0
0.0026	0	0.00548	0.005569	0.005456	0
0.0032	0	0.005466	0.005555	0.005442	0
0.0038	0	0.005459	0.005547	0.005435	0
0.0044	0	0.005459	0.005547	0.005436	0
0.005	0	0.005468	0.005556	0.005445	0

## Appendix A

More details about the coefficients $A_{i}\left\{i=1\ldots 52\right\}$ and $B_{i}\left\{i=1\ldots 5\right\}$ in Equations (19)–(22) are reported below:

$A_{1}=\int_{\frac{-h}{2}}^{\frac{h}{2}}rQ_{11}dz$	$A_{2}=\int_{\frac{-h}{2}}^{\frac{h}{2}}rzQ_{11}dz$
$A_{3}=\int_{\frac{-h}{2}}^{\frac{h}{2}}rz^{2}Q_{11}dz$	$A_{4}=\int_{\frac{-h}{2}}^{\frac{h}{2}}rz^{3}Q_{11}dz$
$A_{5}=\int_{\frac{-h}{2}}^{\frac{h}{2}}rz^{4}Q_{11}dz$	$A_{6}=\int_{\frac{-h}{2}}^{\frac{h}{2}}rz^{5}Q_{11}dz$
$A_{7}=\int_{\frac{-h}{2}}^{\frac{h}{2}}rz^{6}Q_{11}dz$	$A_{8}=\int_{\frac{-h}{2}}^{\frac{h}{2}}rQ_{55}dz$
$A_{9}=\int_{\frac{-h}{2}}^{\frac{h}{2}}rzQ_{55}dz$	$A_{10}=\int_{\frac{-h}{2}}^{\frac{h}{2}}rz^{2}Q_{55}dz$
$A_{11}=\int_{\frac{-h}{2}}^{\frac{h}{2}}rz^{3}Q_{55}dz$	$A_{12}=\int_{\frac{-h}{2}}^{\frac{h}{2}}rz^{4}Q_{55}dz$
$A_{13}=\int_{\frac{-h}{2}}^{\frac{h}{2}}rz^{5}Q_{55}dz$	$A_{14}=\int_{\frac{-h}{2}}^{\frac{h}{2}}rz^{6}Q_{55}dz$
$A_{15}=\int_{\frac{-h}{2}}^{\frac{h}{2}}Q_{12}dz$	$A_{16}=\int_{\frac{-h}{2}}^{\frac{h}{2}}zQ_{12}dz$
$A_{17}=\int_{\frac{-h}{2}}^{\frac{h}{2}}z^{2}Q_{12}dz$	$A_{18}=\int_{\frac{-h}{2}}^{\frac{h}{2}}z^{3}Q_{12}dz$
$A_{19}=\int_{\frac{-h}{2}}^{\frac{h}{2}}z^{4}Q_{12}dz$	$A_{20}=\int_{\frac{-h}{2}}^{\frac{h}{2}}z^{5}Q_{12}dz$
$A_{21}=\int_{\frac{-h}{2}}^{\frac{h}{2}}z^{6}Q_{12}dz$	$A_{22}=\int_{\frac{-h}{2}}^{\frac{h}{2}}rQ_{13}dz$
$A_{23}=\int_{\frac{-h}{2}}^{\frac{h}{2}}\left(r*z*Q_{13}\right)dz$	$A_{24}=\int_{\frac{-h}{2}}^{\frac{h}{2}}rz^{2}Q_{13}dz$
$A_{25}=\int_{\frac{-h}{2}}^{\frac{h}{2}}rz^{3}Q_{13}dz$	$A_{26}=\int_{\frac{-h}{2}}^{\frac{h}{2}}rz^{4}Q_{13}dz$
$A_{27}=\int_{\frac{-h}{2}}^{\frac{h}{2}}(rz^{5}Q_{13}dz$	$A_{28}=\int_{\frac{-h}{2}}^{\frac{h}{2}}\frac{Q_{22}}{r}dz$
$A_{29}=\int_{\frac{-h}{2}}^{\frac{h}{2}}z\frac{Q_{22}}{r}dz$	$A_{30}=\int_{\frac{-h}{2}}^{\frac{h}{2}}z^{2}\frac{Q_{22}}{r}dz$
$A_{31}=\int_{\frac{-h}{2}}^{\frac{h}{2}}z^{3}\frac{Q_{22}}{r}dz$	$A_{32}=\int_{\frac{-h}{2}}^{\frac{h}{2}}z^{4}\frac{Q_{22}}{r}dz$
$A_{33}=\int_{\frac{-h}{2}}^{\frac{h}{2}}z^{5}\frac{Q_{22}}{r}dz$	$A_{34}=\int_{\frac{-h}{2}}^{\frac{h}{2}}z^{6}\frac{Q_{22}}{r}dz$
$A_{35}=\int_{\frac{-h}{2}}^{\frac{h}{2}}(Q_{23}dz$	$A_{36}=\int_{\frac{-h}{2}}^{\frac{h}{2}}zQ_{23}dz$
$A_{37}=\int_{\frac{-h}{2}}^{\frac{h}{2}}z^{2}*Q_{23}dz$	$A_{38}=\int_{\frac{-h}{2}}^{\frac{h}{2}}z^{3}Q_{23}dz$
$A_{39}=\int_{\frac{-h}{2}}^{\frac{h}{2}}z^{4}Q_{23}dz$	$A_{40}=\int_{\frac{-h}{2}}^{\frac{h}{2}}(z^{5}Q_{23}dz$
$A_{41}=\int_{\frac{-h}{2}}^{\frac{h}{2}}rQ_{33}dz$	$A_{42}=\int_{\frac{-h}{2}}^{\frac{h}{2}}rzQ_{33}dz$
$A_{43}=\int_{\frac{-h}{2}}^{\frac{h}{2}}rz^{2}Q_{33}dz$	$A_{44}=\int_{\frac{-h}{2}}^{\frac{h}{2}}rz^{3}Q_{33}dz$
$A_{45}=\int_{\frac{-h}{2}}^{\frac{h}{2}}rz^{4}Q_{33}dz$	$A_{46}=\int_{\frac{-h}{2}}^{\frac{h}{2}}\left(-TQ_{12}\alpha_{11}-TQ_{22}\alpha_{22}-TQ_{23}\alpha_{22}\right)dz$
$A_{47}=\int_{\frac{-h}{2}}^{\frac{h}{2}}z\left(-TQ_{12}\alpha_{11}-TQ_{22}\alpha_{22}-TQ_{23}\alpha_{22}\right)dz$	$A_{48}=\int_{\frac{-h}{2}}^{\frac{h}{2}}z^{2}\left(-TQ_{12}\alpha_{11}-TQ_{22}\alpha_{22}-TQ_{23}\alpha_{22}\right)dz$
$A_{49}=\int_{\frac{-h}{2}}^{\frac{h}{2}}z^{3}\left(-TQ_{12}\alpha_{11}-TQ_{22}\alpha_{22}-TQ_{23}\alpha_{22}\right)dz$	$A_{50}=\int_{\frac{-h}{2}}^{\frac{h}{2}}r\left(-TQ_{13}\alpha_{11}-TQ_{23}\alpha_{22}-TQ_{33}\alpha_{22}\right)dz$
$A_{51}=\int_{\frac{-h}{2}}^{\frac{h}{2}}rz\left(-TQ_{13}\alpha_{11}-TQ_{23}\alpha_{22}-TQ_{33}\alpha_{22}\right)dz$	$A_{52}=\int_{\frac{-h}{2}}^{\frac{h}{2}}rz^{2}\left(-TQ_{13}\alpha_{11}-TQ_{23}\alpha_{22}-TQ_{33}\alpha_{22}\right)dz$
$B_{1}=k_{2}\left(\frac{h}{2}+R\right)$	$B_{2}=k_{2}\left(-\frac{h}{2}+R\right)$
$B_{3}=k_{1}\left(\frac{h}{2}+R\right)$	$B_{4}=k_{1}\left(-\frac{h}{2}+R\right)$
$B_{5}=P_{i}\left(-\frac{h}{2}+R\right)$	
